# Effects on communication due to face mask use: an integrative review

**DOI:** 10.1590/0034-7167-2022-0674

**Published:** 2023-10-09

**Authors:** Jonathan Grassi, Isabel Barreto de Oliveira, Laura Franco Chiriboga, Andréa Alves Maia, Margareth Attianezi, Aline Neves Pessoa Almeida

**Affiliations:** I Universidade Federal do Espírito Santo. Vitória, Espírito Santo, Brazil

**Keywords:** Masks, Communication, Auditory Perception, COVID-19, Review., Máscaras, Comunicación, Percepción Auditiva, COVID-19, Revisión., Máscaras, Comunicação, Percepção Auditiva, COVID-19, Revisão.

## Abstract

**Objectives:**

to integrate evidence from studies on auditory perceptual and speech production effects in communication situations with face mask use.

**Methods:**

an integrative literature review, in MEDLINE, Cochrane Library and Embase databases. The guiding question was: what effects on communication (perceptual-auditory and speech production) occur with face mask use?

**Results:**

searches in electronic databases resulted in 1,478 studies and filtering resulted in 29 final studies.

**Conclusions:**

mask use has effects on communication, both in perception and speech production, factors that are also related to quality of life, stress and socio-emotional factors. These data can impact on indicators and alerts in favor of adopting strategies to manage mask use, involving speech production and perception when wearing a mask in health services.

## RESUMO

**Objetivos:**

integrar evidências de estudos sobre efeitos perceptivos auditivos e de produção de fala em situações de comunicação com o uso de máscara facial.

**Métodos:**

revisão integrativa da literatura, nas bases MEDLINE, *Cochrane Library* e Embase. O questionamento direcionador foi: quais efeitos na comunicação (perceptivo-auditivos e de produção de fala) ocorrem com o uso de máscaras faciais?

**Resultados:**

as buscas nas bases de dados eletrônicas resultaram em 1.478 estudos, e a filtragem culminou em 29 estudos finais.

**Conclusões:**

o uso de máscaras traz efeitos na comunicação, tanto na percepção quanto na produção da fala, fatores ademais relacionados à qualidade de vida, estresse e socioemocionais. Esses dados podem impactar em indicadores e alertas em prol da adoção de estratégias ao manejo de uso de máscaras, envolvendo a produção e percepção de fala em ocasião de uso de máscara nos serviços de saúde.

## RESUMEN

**Objetivos:**

integrar evidencias de estudios sobre efectos perceptuales auditivos y de producción del habla en situaciones de comunicación con el uso de mascarilla facial.

**Métodos:**

revisión integrativa de la literatura, en las bases de datos MEDLINE, Cochrane Library y Embase. La pregunta orientadora fue: ¿qué efectos en la comunicación (perceptivo-auditiva y producción del habla) se producen con el uso de mascarillas?

**Resultados:**

las búsquedas en bases de datos electrónicas dieron como resultado 1.478 estudios y el filtrado dio como resultado 29 estudios finales.

**Conclusiones:**

el uso de mascarillas tiene efectos en la comunicación, tanto en la percepción como en la producción del habla, factores que también se relacionan con la calidad de vida, el estrés y factores socioemocionales. Estos datos pueden impactar en indicadores y alertas a favor de la adopción de estrategias para gestionar el uso de mascarillas, involucrando la producción y percepción del habla al usar mascarilla en los servicios de salud.

## INTRODUCTION

It is known that the pandemic caused by COVID-19, a disease caused by the SARS-CoV-2 virus (hereinafter COVID-19) infection, has strongly affected the way people communicate. The high rate of virus dispersion occurs through aerosols (respiratory droplets), identified as a striking cause of infection when in contact with mucous membranes (nose and mouth) or conjunctivae (eyes), through breathing, talking, coughing or sneezing. who are infected with the virus^(1)^.

One of the measures recommended by the World Health Organization (WHO) for disease prevention and control is mask use, which, together with physical distancing, respiratory etiquette and hand hygiene, aims to interrupt the COVID-19 cycle. Such measures changed interpersonal communication conditions, since, in the pandemic’s critical period, people, in all public spaces and health environments, started to use masks as a public health intervention for measures to protect the general population. Exceptions to this recommendation were observed in children under 5 years old, where mask use was not mandatory, and in children between 5 and 11 years old, in which the decision to wear a mask should be made taking into account adequate adult supervision and the possibility of any potential impact on learning and psychosocial development^(1-2)^.

Although the masks’ primary function is known, we are faced with the implications of their use in communication. Studies have shown the impacts on the auditory-perceptual conditions of the interlocutor who perceives communication through speech reading and acoustic conditions from the filter and the speech production conditions of the interlocutor who uses a mask, which has brought peculiarities to speech production such as vocal quality^(3-4)^.

The main types of masks for preventing disease transmission are fabric (protection for non-professional use), surgical masks and face masks with an N95 filter (respiratory protection equipment). Fabric masks are indicated for collective extra-domestic environments, especially in public transport and at events and meetings. These non-hospital face masks do not offer total protection against infections, but they reduce their incidence when their composition strictly follows the Brazilian National Health Regulatory Agency (ANVISA - *Agência Nacional de Vigilância Sanitária*) recommendations of having at least two layers of cloth, such as cotton or tricoline or TNT^(2)^. The Ministry of Health recommends that surgical and N95/PFF2 masks (for professional use) be prioritized for health professionals, considering that health services are the places that contain the greatest potential for the concentration of viruses and by other groups of workers for whom there is a legal provision for using this personal protective equipment (PPE)^(5)^.

The main guidelines for using face masks ensure that they fully cover the mouth and nose and that they are well adjusted to the face. Due to the need for sealing for individual protection, mask use had an impact on communication due to changes in vocal signal and speech intelligibility quality^(6)^.

Depending on the type of mask and the environment (noise), the effects on speech signal occur differently. Moreover, it prevents the visualization of interlocutors’ face, creating a visual perceptual barrier (in addition to the auditory perceptual one) of communication information (speech reading) during communication. This barrier alone can already be considered harmful to speech perception, since the middle and lower thirds of the face are very influential in emotional recognition^(7)^, and this block impacts the communicated message, especially in noisy environments or when the subject has a hearing impairment^(8)^. It is known that non-verbal communication, such as gestures and facial expressions, constitutes 55% of general communication^(9)^. Therefore, from the impediment of the expressiveness that is generated by phonoarticulatory organ movement, the tendency is that there is an increase in vocal intensity in an attempt to compensate for the difficulty of feedback, because with muffled speech, there is a decrease in the perception of one’s own voice and, consequently, greater effort of the vocal tract, causing wear and tension^(10)^.

Due to the aforementioned considerations, this study is justified and has an impact on speech therapy in favor of communication strategies in new communication situations experienced due to face mask use.

## OBJECTIVES

To integrate evidence from studies on auditory perceptual and speech production effects in communication situations with face mask use.

## METHODS

### Ethical aspects

As this is an integrative review, the research is exempt from the need for submission and approval by the Research Ethics Committee.

### Study design

This study is an integrative literature review, which is used to synthesize and integrate current evidence in a specific area, when published studies have a variety of designs to address a specific problem^(11)^. In order to guarantee data reliability and methodological transparency of this review, the study was registered in Open Science Framework doi.org/10.17605/OSF.IO/WCKBV.

The research question was elaborated using the acronym PECOS^(12)^ (Patient/Population, Intervention/Exposure, Control or Comparison, Outcomes, Study Design), where P = Population (young adults and adults ≥ 18 years old of both sexes, of any ethnicity), I = Intervention (mask use), O = Outcomes (voice quality, auditory perception, speech acoustics), S = Study designs (all studies with quantitative approaches - descriptive, observational and experimental studies). This strategy facilitated the structuring of critical thinking on the subject and the formulation of the following question: what scientific evidence is available from observational and experimental studies on the impact of using a face mask on communication in view of data on auditory-perceptual conditions, vocal quality and speech production in adult interlocutors? Integrative reviews include diverse data sources that enhance a holistic understanding of issues relevant to health care and policy. Thus, the following steps were used for a more systematic and rigorous approach to the process: problem identification; literature search; data assessment; data analysis; and finally presentation of results^(13)^.

### Study period and place

The search for evidence available in the literature was carried out from December 2021 to February 2022. It covered the Medical Literature Analysis and Retrieval System Online (MEDLINE) via PubMed, Cochrane Library and Excerpta Medica database (Embase) databases. For the search strategy, we included a combination of controlled descriptors (indexers in the respective databases) and keywords. Thus, to search for articles in MEDLINE, we used the controlled descriptors of Medical Subject Headings (MeSH) and the Entree terms for Embase, using Boolean operators AND/OR. According to the Cochrane Collaboration^(14)^, these three bibliographic databases are considered the most important sources for a review. MEDLINE (as of January 2022) contains approximately 30 million references to journal articles in biomedicine and health from 1946 onwards. More than 5,000 journals in about 40 languages are indexed in this database. Embase (as of January 2022) contains over 35 million records from 1974 onwards, including records from over 8,000 currently published journals from approximately 100 countries. As of January 2022, the Cochrane Library contains over 1,800,000 trial report records/trial registries potentially eligible for inclusion in Cochrane Reviews, by far the majority of which are randomized trials^(14)^.

Following the acronym PECOS^(12)^, the search strategy for the respective databases was established (Chart 1).

**Chart 1:** Search strategy in the 3 databases, Vitória, Espírito Santo, Brazil, 2022

MEDLINE/PubMed	POPULATION #1 ((“Young Adult” [MeSH Terms] OR “Adult” [MeSH Terms])) EXPOSURE #2 ((“Masks” [MeSH Terms] OR “Mask” [All Fields] OR “N95 Respirators” [MeSH Terms] OR “N95 Respirator” [All Fields] OR “Respirator, N95” [All Fields] OR “N95 Face Masks” [All Fields] OR “Face Mask, N95” [All Fields] OR “N95 Face Mask” [All Fields] OR “N95 Masks” [All Fields] OR “Mask, N95” [All Fields] OR “N95 Mask” [All Fields] OR “N95 Filtering Facepiece Respirators” [All Fields] OR “N95 FFRs” [All Fields] OR “N95 FFR” [All Fields])) OUTCOMES #3 ((“Voice Quality” [MeSH Terms] OR “Voice Qualities” [All Fields] OR “Auditory Perception” [MeSH Terms] OR “Perception, Auditory” [All Fields] OR “Auditory Processing” [All Fields] OR “Processing, Auditory” [All Fields] OR “Speech Acoustics” [MeSH Terms] OR “Acoustics, Speech” [All Fields] OR “Acoustic, Speech” [All Fields] OR “Speech Acoustic” [All Fields])) #4 #1 AND #2 AND #3
Cochrane Library	POPULATION #1 (Young Adult) OR (Adult) EXPOSURE #2 (Masks) OR (Mask) OR (N95 Respirators) OR (N95 Respirator) OR (Respirator, N95) OR (N95 Face Masks) OR (Face Mask, N95) OR (N95 Face Mask) OR (N95 Masks) OR (Mask, N95) OR (N95 Mask) OR (N95 Filtering Facepiece Respirators) OR (N95 FFRs) OR (N95 FFR) OUTCOMES #3 (Voice Quality) OR (Voice Qualities) OR (Auditory Perception) OR (Perception, Auditory) OR (Auditory Processing) OR (Processing, Auditory) OR (Speech Acoustics) OR (Acoustics, Speech) OR (Acoustic, Speech) OR (Speech Acoustic)
Embase	POPULATION #1 (‘adult’ OR ‘young adult’) EXPOSURE #2 (‘Masks’ OR ‘Mask’ OR ‘Minimally 94 percent efficient filtering facepiece respirator’ OR ‘N-95 Respirator’ OR ‘N95 control respirator’ OR ‘N95 face-mask’ OR ‘N95 facemask’ OR ‘N95 facial mask’ OR ‘N95 FFR’ OR ‘N95 FFRs’ OR ‘N95 filtering face piece’ OR ‘ N95 filtering facepiece’ OR ‘95 filtering facepiece particulate respirator’ OR ‘N95 filtering facepiece respirator’ OR ‘N95 half-mask respirator’ OR ‘N95 mask’ OR ‘N95 particulate filter respirator’ OR ‘N95 particulate filtering facepiece respirator’ OR ‘N95 respirator’ OR ‘N95 respirators’ OR ‘N95 surgical mask respirator’ OR ‘surgical mask’) OUTCOMES #3 (‘Voice’ OR ‘voice quality’ OR ‘vox’ OR ‘auditory processing disorder’ OR ‘auditory comprehension disorder’ OR ‘auditory perception disorder’ OR ‘auditory perceptual disorder’ OR ‘central auditory processing disorder’ OR ‘psychoacoustical disorder’ OR ‘speech’ OR ‘speech acoustics’ OR ‘speech production’)

### Sample

In the initial search in the selected databases, 1,478 studies were found and, after removing duplicates, 1,353 studies remained in the identification stage. After applying the eligibility criteria, 29 studies were selected to compose the results.

### Inclusion and exclusion criteria

Studies with a quantitative approach (observational and experimental), conducted with adults wearing masks, of both sexes were included. It is noteworthy that no date or language restrictions were applied in the selection of studies.

Qualitative, experimental studies conducted in animal models, *in vivo, ex vivo* on this topic were excluded.

### Study selection

The search for studies was carried out by two independent researchers (IB, JGR), carried out at the same time. After the initial search, articles were saved in Endnote Web™ bibliographic software to store, organize, manage all references and identify duplicates. Duplicates were only counted once. Studies were exported to the Rayyan™ application^(15)^, a tool that assists in the screening and selection of studies and provides greater transparency of the method at this stage^(16-17)^. The first phase of the study took place through screening by titles and abstracts and the authors verified whether the study met the inclusion criteria, noting whether the population and intervention are of interest, whether participants do not have any type of health condition that should be excluded, and whether the study design was eligible. A third reviewer (ANPA) resolved disagreements regarding the selected studies.

After the initial screening, phase 2 was followed, in which the same two independent researchers (IB, JGR) evaluated the full text of the retrieved studies for inclusion/exclusion, also using the Rayyan™ application and the third reviewer (ANPA) was convened to resolve disagreements.

Studies selected as eligible in the first selection phase (title and abstract screening) were analyzed in detail. In the same way as in the first selection phase, the authors searched the text for keywords that identified the eligibility criteria in relation to the review’s PECOS concept.

### Data extraction

The studies selected to compose the sample were read in full and analyzed by the researchers, who independently mapped the data for each included study based on previously published forms^(16-18)^. Extracted information included: a) study identification, with data such as article title, journal impact factor, country of study authors, year of publication, study host institution (hospital, university, research center, multicenter study or study at a single institution), conflicts of interest and funding; b) methodological characteristics (study design, study objective, research question or hypotheses, sample characteristics such as sample size), age, baseline characteristics of experimental and control groups, method of recruitment, losses, duration of follow-up and statistical analyses; c) main findings and implications for clinical practice; and d) conclusions.

### Methodological quality assessment

The assessment of studies’ methodological quality was defined as an essential process to establish internal validity, verifying possible biases and the reliability of the identified evidence. For the classification of selected studies, we used the hierarchy of evidence divided into seven levels, commonly used in high impact publications^(19)^, namely: I) evidence from systematic reviews or meta-analyses of randomized controlled clinical trials (RCTs); II) evidence from well-designed RCT; III) evidence from well-designed non-randomized controlled clinical trials (quasi-experimental); IV) evidence from well-designed case-control, cohort, or cross-sectional studies; V) evidence from systematic reviews of qualitative and descriptive studies; VI) evidence from a single descriptive or qualitative study; and VII) evidence from opinion of authorities and/or reports of expert committees.

## RESULTS

Searches in the three electronic databases resulted in 1,478 studies (659 articles in MEDLINE via PubMed; 294 articles in the Cochrane Library; 525 articles in Embase). Filtering culminated in 29 final studies: 1 national study and 28 international studies. Among the 29 articles included, predominantly, studies were developed in 2021 (74.19%, n=23), with cross-sectional observational designs. Cohort, descriptive, comparative, randomized and non-randomized, and prospective studies are also part of the research selected for the integrative review.

**Figure 1:**
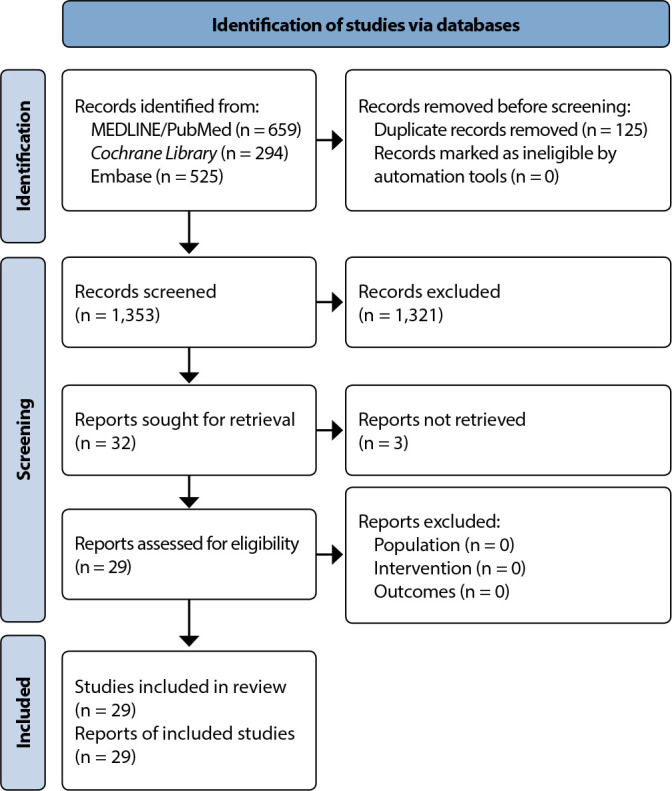
PRISMA flowchart for selecting articles

The main results of included studies were systematized according to the similarities of data analyzed in each research. From this, the following categories were originated for the description and discussion of results: *Effects on voice quality and speech acoustics* (9 studies); *Auditory-perceptual effects* (13 studies); and *Data on personal protective equipment characteristics* (7 studies) (Figure 1).

Charts 2, 3 and 4 chronologically summarize the main characteristics of the studies included in the qualitative synthesis according to axes *Effects on voice quality and speech acoustics*, *Auditory-perceptual effects* and *Data on personal protective equipment characteristics*, respectively.

**Chart 2:** Characteristics of studies that assessed effects on voice quality and speech acoustics included in the integrative review

Title	Country/year	Design/number of patients	Intervention	Outcome	Level of Evidence
Voice Differences When Wearing and Not Wearing a Surgical Mask^(20)^	Italy, 2021	Longitudinal, case-control study n= 60	With intervention of mask use.	Mask use can induce an unconscious need to increase vocal effort, resulting in a greater risk of developing functional dysphonia.	IV
Voice Acoustics and Vocal Effort in Mask-Wearing Healthcare Professionals: A Comparison Preand Post-Workday^(21)^	USA, 2021	Cross-sectional study. n=18	No intervention	Health professionals who use masks reported greater vocal symptoms after working hours compared to pre-workdays.	IV
The Effects of the Use of Protective Face Mask on the Voice and Its Relation to Self-Perceived Voice Changes^(22)^	Greece, 2021	Cross-sectional, observational study n=155	No intervention	Protective face mask use may result in the onset of a voice disorder, particularly in the high-risk population.	IV
The Effect of Masks and Respirators on Acoustic Voice Analysis During the COVID-19 Pandemic^(23)^	Turkey, 2021	Prospective study n=204	With intervention of mask use.	Significant difference only in the Shimmer and HNR values in relation to the other analysis values.	IV
Self-Perceived Voice Handicap During COVID19 Compulsory Facemask Use: A Comparative Study Between Portuguese and Spanish Speakers^(24)^	Spain, 2021	Comparative observational descriptive study n = 558	With intervention of mask use.	Overall VHI scores and all-dimension scores were higher for the masked condition.	IV
Effects of Medical Masks on Voice Assessment During the COVID-19 Pandemic^(25)^	China, 2021	Cross-sectional study n=53 ​​	With intervention of mask use.	Healthy participants showed a significantly higher sound pressure level, less perturbation and a decrease in F3 after using medical masks.	IV
COVID-19: Acoustic Measures of Voice in Individuals Wearing Different Facemasks^(26)^	USA, 2021	Cross-sectional study. n=19	With intervention of mask use.	Masks tested did not have a significant impact on intensity, fundamental frequency. frequency, PPC-s, frequency of the first or second formant compared to the unmasked speech output.	IV
Acoustic voice characteristics with and without wearing a facemask^(27)^	Australia, 2021	Case-control, n=16	With intervention of mask use	The surgical mask has less impact than the KN95 on analyzed vocal aspects	IV
Are Acoustic Markers of Voice and Speech Signals Affected by Nose-and-Mouth-Covering Respiratory Protective Masks?^(28)^	Belgium, 2021	Cross-sectional study n=50	With intervention of mask use	The surgical mask is preferred when spoken communication is a priority alongside respiratory protection.	IV

**Chart 3:** Characteristics of studies that assessed auditory-perceptual effects in the integrative review

Title	Country/year	Design/number of patients	Intervention	Outcome	Level of Evidence
Effects of face masks on acoustic analysis and speech perception: Implications for peri-pandemic protocols^(3)^	Australia, 2020	Cross-sectional study n=7	With intervention of mask use.	Face masks alter the speech signal, but measures of vocal quality remain unchanged.	IV
Impact of face masks in public spaces during COVID-19 pandemic on daily life communication of cochlear implant users^(29)^	Netherlands, 2020	Prospective research study n=221	No intervention.	Face mask use reduces Cochlear Implant (CI) users’ quality of life.	IV
Effect of Wearing a Face Mask on Vocal Self-Perception during a Pandemic^(30)^	Brazil, 2021	Observational, descriptive and cross-sectional study n=468	No intervention.	Face mask use increases the perception of symptoms and vocal discomfort, especially in individuals who used them for professional and essential activities.	IV
Effects of face masks on speech recognition in multi-talker babble noise^(31)^	USA, 2021	Randomized clinical trials n= 200	With intervention of mask use.	Different types of masks show similar accuracy at low background noise levels and more apparent at high noise levels.	II
Face mask type affects audiovisual speech intelligibility and subjective listening effort in young and older adults^(32)^	USA, 2021	Non-randomized controlled trial n=180	With intervention of mask use and environmental noise addition.	Older adults showed worse general intelligibility and classified speech as more difficult to process compared to young adults	III
Impact of Masks on Speech Recognition in Adult Patients with and without Hearing Loss^(33)^	USA, 2021	Case-control n=45	No intervention.	Dramatic decrease in word recognition scores when the provider utters words through an N95 mask and especially when the speaker is a woman. (p < 0.001; 95% CI:10-26%).	IV
Influence of surgical and N95 face masks on speech perception and listening effort in noise^(34)^	Germany, 2021	Prospective, observational study n=17	No intervention.	Face masks reduce speech perception and increase listening effort in different noise signals.	IV
Powered air-purifying respirators used during the SARS-CoV-2 pandemic significantly reduce speech perception^(35)^	Germany, 2021	Cross-sectional study n = 10	No intervention.	The assessed powered air-purifying respirator system can be considered for high-risk procedures in SARS-CoV-2 positive cases in conjunction with a hearing protector.	IV
The cafeteria study: Effects of facial masks, hearing protection, and real-world noise on speech recognition^(36)^	USA, 2021	Cross-sectional study n=34	With intervention of mask use and environmental noise addition.	Speech recognition in real-world listening environments can be hindered by PPE worn by speakers and listeners.	IV
The impact of face masks on the recall of spoken sentences^(37)^	Germany, 2021	Non-randomized controlled trial n=32	No intervention.	Listeners remembered significantly fewer words when the phrases were spoken with a face mask.	III
Influence of Protective Face Coverings on the Speech Recognition of Cochlear Implant Patients^(38)^	USA, 2021	Prospective cohort study n=23	No intervention.	The type and combination of protective face coverings used have differential effects on the attenuation of speech information, influencing the speech recognition of patients with hearing loss.	IV
Communication with face masks during the COVID-19 pandemic for adults with hearing loss^(39)^	Canada, 2022	Cross-sectional study n=656	No intervention.	Increased public awareness and use of a transparent mask were determined to be examples of practical supports for effective social interaction.	IV
How Face Masks Interfere With Speech Understanding of Normal-Hearing Individuals: Vision Makes the Difference^(40)^	Germany, 2022	Prospective cohort study n=15	Different experimental conditions with and without simulated face masks using the audio-visual version of the female German matrix test.	Face mask use by the speaker leads to a deterioration in speech understanding by the listener.	IV

**Chart 4:** Characteristics of studies that assessed data on personal protective equipment characteristics in the integrative review

Authors	Country/year	Design/number of patients	Intervention	Outcome	Level of Evidence
The effects of N95 mask and face shield on speech perception among healthcare workers in the coronavirus disease 2019 pandemic scenario^(41)^	India, 2020	Prospective observational study n=20	No intervention.	PPE use significantly impairs speech perception. Increased speech reception threshold (mean of 12.4 dB) and decrease in speech discrimination score (mean of 7%).	IV
Acoustic voice analysis in the COVID-19 era^(42)^	Italy, 2020	Cross-sectional study n=50	With intervention of mask use.	None of the variations in the acoustic analysis of the voice detected wearing a surgical mask and not wearing a surgical mask was statistically significant.	IV
Association of In-Ear Device Use With Communication Quality Among Individuals Wearing Personal Protective Equipment in a Simulated Operating Room^(43)^	Canada, 2021	Clinical trial n=12	With intervention of mask use and addition of in-ear device.	New in-ear device associated with better communication and reduced listening effort for healthcare professionals in the operating room.	IV
Impact of Face Masks on Speech Acoustics and Vocal Effort in Healthcare Professionals^(44)^	USA, 2022	Quasi-experimental and between-subject design n=21	With intervention of mask use.	Face masks represent an additional barrier to effective communication that mainly affects spectral characteristics, vowel space measurements and vocal effort.	III
Prevalence of Voice Disorders in Healthcare Workers in the Universal Masking COVID-19 Era^(45)^	Chile, 2021	Cross-sectional study n=218	No intervention.	Health workers in high-risk hospital units during the COVID-19 pandemic are at risk of vocal disorders.	IV
The Impact of Masking Habits on Voice in a Subpopulation of Healthcare Workers^(46)^	Lebanon and USA, 2022	Cross-sectional study n=178	No intervention.	Mask use during the COVID-19 pandemic was associated with a high prevalence of fatigue, exertion, and abnormal VHI-10 scores.	IV
Acoustic characteristics of fricatives, amplitude of formants and clarity of speech produced without and with a medical mask^(47)^	Australia, 2022	Cross-sectional study n=16	With intervention of mask use.	The root mean square amplitude of all included fricatives was significantly lower in surgical mask and KN95 mask compared to the unmasked condition.	IV

## DISCUSSION

This integrative review intended to synthesize and integrate evidence from studies that discuss data on auditory-perceptual and speech production effects in communication situations with face mask use. In summary, among the 29 studies that met all the inclusion criteria, 9 addressed effects on voice quality and speech acoustics, 13 on auditory-perceptual effects and 7 articles on data on PPE characteristics.

### Effects on voice quality and speech acoustics

Based on studies that aimed to compare the acoustic voice parameters analyzed between the ‘masked’ and ‘unmasked’ speech conditions, it is understood that face mask use influences the significant increase in effort and vocal fatigue^(20,22,24-25,45)^.

Authors argue that there are differences in the vocal effects caused between different types of masks^(26,28)^. In speech/voice production, studies worldwide have shown that face mask use impacts the distribution of energy at frequencies above 3 kHz for a N95 mask and above 5 kHz for surgical and cloth masks^(3)^. Surgical and N95 masks can attenuate high frequency sounds between 3 and 12 dB^(48-49)^. Therefore, surgical masks are those that interfere less in individuals’ vocal quality, when compared to N95^(25-28,44,47)^, containing minimally significant effects when compared to PPE of the type that offer respirators that mainly interfere with vocal intensity, signal-to-noise ratio (SNR), smoothed cepstral peak prominence (PPC-s) and fundamental voice frequency^(21,23,25-26,44)^. In addition to this, face masks increased the difficulty in speech intelligibility and intensified pneumophonic incoordination^(22-23,30,33)^.

### Auditory-perceptual effects

As for the effects of face masks on speech perception, the authors of selected studies state that there is a change in speech signal, its discrimination and its intelligibility in terms of word production accuracy, together with the difficulty in reading speech, due to the impediment of viewing the middle third of the face, which impairs the understanding of what was communicated and expressed^(33-34)^.

Speech understanding with mask use is significantly worse than under control conditions without masks^(23)^, and speech intelligibility in SNR is affected by an average of 4.1 dB when simulating a surgical mask and by 5.1 dB when simulating a cloth mask. Furthermore, face mask use by the speaker leads to a decrease in speech understanding by normal listeners^(27,33)^. Similarly, the data indicate that the speech made using a face mask requires a greater auditory effort, mainly from older adults, and that there is a reduction in quality of life due to impairments in speech signal and in the daily communication of individuals with hearing loss, such as CI users^(32)^. The average auditory recognition of words shows a gradual decrease in the score with surgical mask and with N95 masks. In subjects with self-reported hearing loss, mean word recognition scores reached 46% with an N95 mask compared to 79% in patients who reported normal hearing (p < 0.001)^(31)^.

Questionnaires applied to CI users demonstrate the worsening of communication intelligibility in the reception of speech sounds coming from individuals who use face masks, and authors believe that the alterations would be similar for other groups of individuals with and without significant hearing loss^(29,38)^.

### Data on personal protective equipment characteristics

Studies indicate that individuals who use a face mask are at risk of developing vocal disorders due to the combination of factors such as working hours, especially those who use surgical masks^(30,45)^ and especially when a face shield is used simultaneously.

In an analysis to compare differences between sex and six different mask conditions (no mask, cloth mask, surgical mask, N95 mask, and surgical mask over an N95 mask with and without face shield) for measures of voice intensity and acoustic variables, it was found that tested masks did not have a significant impact on loudness, fundamental frequency, PPC-s compared to the unmasked voice output; however, using a face shield affected vocal intensity and PPC-s^(46)^.

Health professionals reported a significant increase in vocal effort after the workday^(21)^. The main parameter adopted was the increase in intensity after the working day compared to the pre-workday, as well as an increase in SNR. On the other hand, there was a decrease in the displacement of relative fundamental frequency in speech production of these health professionals. Studies have applied acoustic voice analysis with definition of parameters such as minimum and maximum pitch in situations with and without surgical mask and number of pulses, periods and speech SNR values and concluded that wearing or not wearing a surgical mask did not significantly affect voice acoustics. Acoustic characteristics of voice quality of people with face mask remain unchanged, regardless of mask type^(3,42)^.

In the search for greater communicative effectiveness, in situations where the interlocutors need to use face masks, studies point to alternative strategies^(41,50)^. For instance, there is using digital platforms for teleconsultations, when internet access is possible, together with using live subtitles, adoption of a greater range of articulatory speech movements, both in online and face-to-face communicative situations^(39)^.

As seen in several studies, the mask has less influence on speech intelligibility when the communicative situation occurs in quiet environments, but there is deterioration in speech perception and understanding with environmental noise addition^(20,31)^.

### Study limitations

A limitation of our study to be considered is that some of the studies included in this review have a cross-sectional design. Such studies are not able to detect differences in auditory perceptual effects and speech production in communication situations with a comparator group and this may have reflected in the conclusion of these studies.

Many studies included in this review take as parameters the characteristics of speech sounds from other languages, mainly English. This fact shows the impact of developing new studies that bring details specifically about filter (mask) characteristics in speech signal considering Brazilian Portuguese (BP), since the acoustic-phonetic characteristics of these speech sounds produced with and without face mask use may change according to language. In this way, the demand for more studies on its effects on communication that are perceived daily during conversations in different acoustic environments of BP speakers is evident. Likewise, data on which managements to prepare the team for strategies in the face of communication difficulties with a face mask are fundamental in understanding this phenomenon, which the filtering of this study may not have contemplated.

### Contributions to health and public policies

Even if it is considered something complex and challenging, the combination of data, in the case of the integrative review, by inserting a systematic analysis of different variables, was organized into thematic axes that allowed reflections on the effects of using masks in communication both in perception and in speech production. Current factors that are also related, such as impacts on quality of life, stress and socio-emotional issues, need to be properly managed in health and education actions by health professionals, interdisciplinary, in different communicative contexts of these conjunctures, especially in services that will continue to adopt the mentioned preventive measure of disease transmission. These data can impact on indicators and alerts in favor of adopting strategies to manage mask use.

Furthermore, environmental acoustics (noise) characteristics in communication must be considered in addition to the effects on speech signal, which are related and interfered differently, according to content specificities. Moreover, public policies on health workers’ vocal and general health (tension, stress, quality of life and others) are highlighted, especially health professionals on working hours and in these communication conditions.

## CONCLUSIONS

It certifies the relevance of developing research on a topic present in world society, arising from mask use since 2020, as a result of the pandemic caused by COVID-19. Mask use has become a practice that will not be extinguished promptly, as it has become essential equipment for those with other respiratory conditions or flu conditions. In this way, the demand for more studies on its effects on communication that are perceived daily during conversations in different environments and types of face mask is highlighted.

The synthesized and analyzed evidence points out that mask use, despite being a primordial measure in disease transmission control, affects communication, such as in speech production, in users’ vocal health, in addition to interfering with understanding the messages issued by interlocutors and affecting quality of life, due to interference in everyday conversations and especially during health services as well as the time of daily use in the working day. In this regard, using facilitating measures in the management of this new reality is strongly recommended.
